# Hypoxia-Inducible Factor-1α Modulates the Toll-Like Receptor 4/Nuclear Factor Kappa B Signaling Pathway in Experimental Necrotizing Enterocolitis

**DOI:** 10.1155/mi/4811500

**Published:** 2024-12-17

**Authors:** Yunfei Zhang, Mei Yan, Yingbin Yue, Yongfeng Cheng

**Affiliations:** Department of Pediatrics, The First Affiliated Hospital of Xinjiang Medical University, Urumqi, Xinjiang 830054, China

**Keywords:** hypoxia-inducible factor-1α, inflammation, necrotizing enterocolitis, NF-κB, oxidative stress

## Abstract

Necrotizing enterocolitis (NEC) is a devastating disease observed in premature infants, characterized by intestinal ischemia and inflammation. Hypoxia-inducible factor-1 alpha (HIF-1α), a master regulator of the cellular response to hypoxia and ischemia, plays a critical role in NEC pathogenesis. However, the precise mechanisms by which HIF-1α influences the intestines in NEC remain poorly understood. Herein, we aimed to explore the role of HIF-1α in NEC using a transgenic mouse model. We induced NEC in neonatal mice from postnatal day 5 to 9, and various parameters, including intestinal injury, oxidative stress, inflammatory responses, intestinal epithelial cell (IEC) proliferation, and apoptosis, were assessed. The results confirmed that the absence of intestinal epithelial HIF-1α increased the susceptibility of mice to NEC-induced intestinal injury, as evidenced by increased oxidative stress, inflammatory responses, apoptosis, and inhibition of proliferation. Additionally, we observed an upregulation of the Toll-like receptor 4 (TLR4)/nuclear factor kappa B (NF-κB) signaling pathway specifically in the intestines of mice lacking HIF-1α in IECs (HIF-1α^ΔIEC^) with NEC. These findings provide crucial insights into the role of HIF-1α in regulating intestinal oxidative stress and inflammation to maintain intestinal homeostasis, highlighting its association with the TLR4–NF-κB signaling pathway. Furthermore, these insights might lead to the identification of novel therapeutic targets for the treatment of NEC.

## 1. Introduction

Necrotizing enterocolitis (NEC), a common intestinal inflammatory disease, is a significant cause of morbidity and mortality, which predominantly affects premature infants [1]. The exact mechanisms contributing to NEC are multifactorial, encompassing immaturity of the gut, hypoxia–ischemia, formula feeding, and microbial dysbiosis [2]. The key initiating events include intestinal microvascular disruption and hypoxia, leading to bacterial translocation across the epithelial tissue, thereby triggering an innate immune response [3]. Ischemic features, such as hemorrhage, edema, mucosal ulceration, vascular congestion, and coagulative necrosis, are evident in histological specimens of NEC [4]. Animal models support the critical role of hypoxia, with the induction of NEC achieved through hypoxia and enteral feeding in neonatal mouse pups [5]. Moreover, a study using two crucial regulators of intestinal microcirculation, endothelin-1 (a vasoconstriction factor) and nitric oxide (NO) (a vasodilation factor), further emphasized the influence of ischemia in NEC pathogenesis [3]. In that study, endothelial NO synthase (eNOS) ablation resulted in early onset and increased severity of NEC in murine models. These results highlight the significance of hypoxia in the pathogenesis of NEC.

Hypoxia-inducible factor-1 (HIF-1) is a highly conserved transcription factor that plays a crucial role in regulating the cellular response to hypoxia and ischemia [6]. All mammals possess this factor, which is essential for the maintenance of oxygen homeostasis [7]. Under normoxic conditions, the α subunit of HIF-1 is regulated by ubiquitin-dependent proteasomal degradation. However, when exposed to hypoxia, the HIF-1α subunit is stabilized and forms a heterodimer with HIF-1β. Once translocated into the nucleus, it binds to HIF response elements, activating the transcription of genes involved in multiple processes, such as erythropoiesis, angiogenesis, metabolic reprogramming, cell proliferation, and apoptosis/survival [8].

The intestines play a critical role as a barrier between the external environment and the internal bloodstream, and the intestinal mucosa is subjected to abrupt changes in blood flow and oxygenation during physiological processes. Consequently, intestinal epithelial cells (IECs) exhibit an innate resistance to hypoxia. They employ basal HIF-1α regulation to cope with hypoxia [9]. Studies have revealed that HIF-1α exerts a protective function in the intestines by regulating gene expression pathways that control oxygen homeostasis [10, 11].

Mice with disrupted HIF-1α specifically in IECs or with knockdown of *Hif1a* demonstrate severe mucosal barrier defects, highlighting the significance of HIF-1α in maintaining barrier integrity [12]. The pathogenesis of NEC is closely associated with an impaired intestinal barrier [1], further emphasizing the importance of understanding the role of HIF-1α in NEC pathogenesis. While the exact signaling mechanisms of HIF-1α in NEC are not yet fully understood, research continues to unravel the complex relationship between hypoxia, HIF-1α activation, and the integrity of the intestinal barrier.

In the current study, we hypothesized that intestinal epithelial HIF-1α plays a critical role in determining the risk of NEC. We explored the underlying mechanisms responsible for HIF-1α's involvement in NEC pathogenesis using an intestinal epithelium conditional knockout mouse model. Our initial findings demonstrated that mice lacking HIF-1α exhibit increased severity of intestinal damage, elevated oxidative stress, cell apoptosis, and persistent inflammation in NEC. These results provide compelling evidence supporting the hypothesis that intestinal epithelial HIF-1α is a crucial regulator of NEC risk. Exploring the details of the signaling mechanisms underlying HIF-1α's activation and its impact on intestinal barrier integrity in NEC might offer valuable insights for the development of novel therapeutic strategies against NEC.

## 2. Materials and Methods

Further details of the methods are shown in the Supporting information.

### 2.1. Animals

We obtained HIF-1α^flox/flox^ mice and Vil1^cre+^ mice from Prof. Chunbao Guo (Chongqing Medical University, Chongqing, China), which were originally purchased from Shanghai Model Organisms, Inc. (Shanghai, China). The mice used in the study had a C57BL/6J background. The present experimental protocol was followed with the guidelines for laboratory animal management and was approved by the institutional review board of Xinjiang medical university. The littermates that did not carry the Vil1^cre+^ transgene were used as controls. A group of mice (half male and half female) were randomly assigned to each experimental group. The control mice were breastfeed by their mothers, while the experimental pups (of various sexes) were isolated on postnatal day 5 (P5) for NEC induction. The isolated pups (body weight: 3–4 g) were housed in 37°C incubators with a 12:12-h dark/light cycle and provided with bedding. NEC was induced in pups from P5 to P9 using lipopolysaccharide (LPS) (4 mg/kg), hypoxia, and hyperosmolar formula, as described previously [13]. On P9, we sacrificed the mice and collected terminal ileum specimens for examination.

### 2.2. Glutathione (GSH) and Oxidized Glutathione (GSSG) Detection

We quantified the production of GSSG and reduced GSH using a GSH and GSSG Assay Kit (S0053, Beyotime, Shanghai, China).

### 2.3. Morphological Assessment

We sectioned the terminal ileal specimens at 4-μm intervals for hematoxylin and eosin (H&E) staining. Two researchers, who were blinded to the treatment groups, used a standard histological scoring system to assess the intestinal injury, which was graded on a 5-point scale (grade 0 to grade 4). Grade 2 or above was defined successful NEC [14].

### 2.4. IEC Isolation and Culture

Primary IECs were isolated and cultured from the resected mouse small intestines as described previously [15]. Briefly, the resected small intestines were washed using sterile medium, incubated in an ethylenediaminetetraacetic acid solution, and then incubated with collagenase type IV. The dissociated cells were washed and incubated at 32°C with 5% CO_2_. The IEC cultures were confirmed using immunological methods, as described previously [16].

#### 2.4.1. Flow Cytometry

Flow cytometry analysis was performed by preparing single-cell suspensions of cultured IECs at a concentration of 1 × 10^6^ cells/mL. Fluorescein isothiocyanate (FITC)–annexin V and propidium iodide (PI) staining from a Cell Apoptosis Kit (BB4101, Bestbio, Shanghai, China) was used to analyze cell apoptosis.

### 2.5. Western Blotting

Intestinal tissues were lysed in radioimmunoprecipitation assay lysis buffer. Equal amounts of protein from all samples were separated by sodium dodecyl sulfate–polyacrylamide gel electrophoresis and transferred to polyvinylidene fluoride membranes. After blocking nonspecific binding, the membranes were incubated with the indicated primary antibodies. Horseradish peroxidase-conjugated secondary antibodies were then used, and the immunoreactive protein bands were visualized and quantified using ImageJ software (NIH, Bethesda, MD, USA). All the primary antibodies are listed in Table S1.

### 2.6. Enzyme-Linked Immunosorbent Assay (ELISA)

The levels of granulocyte macrophage-colony stimulating factor (GM-CSF), C-C motif chemokine ligand 20 (CCL-20), secretory immunoglobulin A (SIgA), β-defensin2, and proinflammatory cytokines in the intestines were detected using ELISA kits. All the ELISA kits are listed in Table S1.

### 2.7. Superoxide Anion Assessment

Superoxide anion levels were assessed using the luminescent O_2_^•–^ reagent lucigenin (CS1000, Sigma, St. Louis, MO, USA). Supernatants from digested mouse tissues were incubated with lucigenin, and luminescence was detected using a luminometer. The results are presented as relative light units (RLU)/mg protein.

### 2.8. Reactive Oxygen Species (ROS) Production Assessment

Protein concentrations in homogenized intestines were measured using the bicinchoninic acid method. The levels of H_2_O_2_ in the intestines were detected by adding respiratory substrates and Amplex Red Reagent (A12222, Invitrogen, Carlsbad, CA, USA), followed by incubation and detection [17].

### 2.9. Superoxide Dismutase (SOD) and Malondialdehyde (MDA) Detection

Mouse intestines were collected and processed via homogenization and centrifugation. The concentrations of SOD and MDA in the supernatants were measured using kits (Nanjing Jiancheng, Nanjing, China) [18].

### 2.10. Intestinal Permeability

Following the guidelines for assessing intestinal permeability [18], we conducted an experiment in which FITC-conjugated dextran (D1844, Invitrogen) was administered intragastrically to a group of pups at 40 mg/100 g body weight. This administration lasted for 4 h. Afterward, the pups were sacrificed, and blood samples were collected to determine the levels of fluorescence in the serum.

### 2.11. Proliferation of IECs

To assess the proliferation of IECs, the mice received an intraperitoneal injection of bromodeoxyuridine (BrdU). After 18 h, the pups were sacrificed, and the terminal ileum was subjected to immunofluorescence staining for BrdU according to standard quantification methods [19].

### 2.12. Quantitative Real-Time Reverse Transcription Polymerase Chain Reaction (RT-qPCR)

An RNA assay kit (AG21024, AG, Hunan, China) was used to extract RNA from mouse intestines. The RNA concentration was assessed, and then the RNA was reverse transcribed to complementary DNA (cDNA). Quantitative amplification of the cDNA was performed using SYBR Premix Ex Taq (AG11708, AG, Hunan). Relative messenger RNA (mRNA) expression was normalized using the ΔΔCq method with *Actb* (encoding β-actin) gene expression as the normalization control. Table S2 provides the primer sequences.

### 2.13. Statistical

GraphPad Prism software (version 8; GraphPad Inc., La Jolla, CA, USA) was used for data processing. The results are presented as the mean ± the standard error of the mean (SEM), assuming a normal distribution of the data. Statistical comparisons were conducted using one-way analysis of variance (ANOVA) with post hoc Tukey analysis or Student's *t*-test, as appropriate. Survival curves were analyzed using the log-rank test. Statistical significance was considered for *p* values less than 0.05.

## 3. Results

### 3.1. Exacerbated Intestinal Damage in HIF-1α^ΔIEC^ Mice Subjected to NEC Stress

To investigate the role of HIF-1α in NEC stress, we generated mice with *Hif1a* knockout specifically in IECs by mating female HIF-1α^flox/flox^ mice with male Vil1^cre+^ mice. The offspring were genotyped using PCR, with mice carrying the genotype HIF1α^flox/flox^/Vil1^cre+^ designated as HIF-1α^ΔIEC^, while their HIF-1α^flox/flox^/Vil1^cre−^ littermates served as wild-type (HIF-1α^loxP/loxP^) controls. Western blotting analysis confirmed the absence of HIF-1α protein expression in the intestinal tissues of the HIF1α^ΔIEC^ mice (Figure S1A,B).

**Figure 1:**
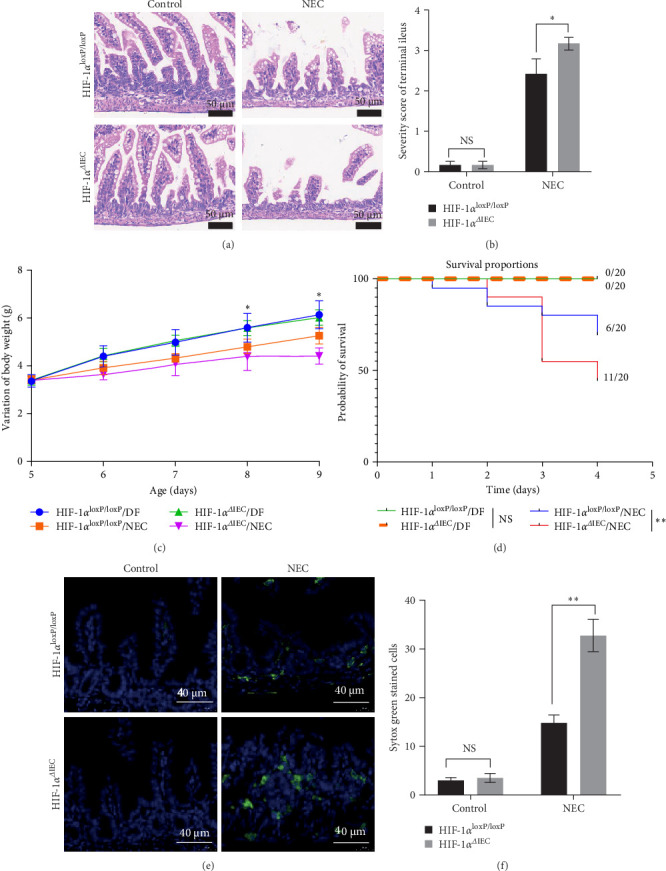
Evaluation of experimental necrotizing enterocolitis (NEC) severity. (A) Hematoxylin and eosin (H&E) staining of terminal ileum specimens. Scale bars: 50 μm. (B) Severity scores based on morphological changes. (C) Body weight changes. (D) Survival rates compared using the Kaplan–Meier method with the log-rank test. (E) Detection of cell death using the Sytox Green necrosis marker. Scale bars: 40 μm. (F) Comparison of necrotic cells between the groups. Two-sided one-way one-way analysis of variance (ANOVA) was utilized for data comparison together with a post hoc Tukey test (*n* = 8–20, means ± the standard error of the mean [SEM]). *⁣*^*∗*^*p* < 0.05, *⁣*^*∗∗*^*p* < 0.001. DF, dam feed; HIF-1α, hypoxia-inducible factor-1 alpha; NS, no statistical difference.

To assess the severity of NEC, we evaluated morphological changes, body weights, and survival rates. The HIF-1α^ΔIEC^ mice exhibited more pronounced morbidity under NEC stress compared with the HIF-1α^loxP/loxP^ mice (Figure 1A–D, Figure S1C). Furthermore, treatment with dimethyloxalylglycine (DMOG), a prolyl hydroxylase domain (PHD) inhibitor that stabilizes HIF-1α, alleviated NEC symptoms in HIF-1α^loxP/loxP^ mice but had no effect on HIF-1α^ΔIEC^ mice (Figure S2A–D). Intestinal cell necrosis, as indicated by Sytox Green staining, a marker to detect dead cells, was increased in the HIF-1α^ΔIEC^ mice compared with that in the HIF-1α^loxP/loxP^ mice under NEC stress (Figure 1E, F). Similarly, DMOG treatment reduced the number of dead intestinal cells only in the HIF-1α^loxP/loxP^ mice (Figure S1E,F).

### 3.2. Enhanced Intestinal Oxidative Stress in HIF-1α^ΔIEC^ Mice Under NEC

**Figure 2:**
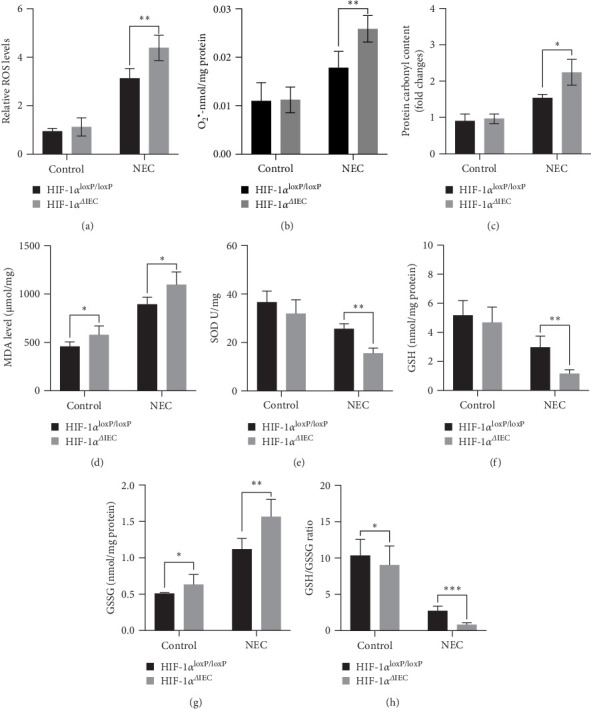
Evaluation of oxidative stress: (A–C) comparison of reactive oxygen species (ROS), superoxide (O_2_^•–^), and carbonyl protein levels between the groups, (D, E) measurement of malondialdehyde (MDA) and superoxide dismutase (SOD) levels in intestinal tissues, and (F–H) evaluation of oxidized glutathione (GSSG) and glutathione (GSH) in the intestines. Data represent three separate experiments. Two-sided one-way one-way analysis of variance (ANOVA) was utilized for data comparison together with a post hoc Tukey test (*n* = 6–8, means ± the standard error of the mean [SEM]). *⁣*^*∗*^*p* < 0.05, *⁣*^*∗∗*^*p* < 0.01, *⁣*^*∗∗∗*^*p* < 0.001. HIF-1α, hypoxia-inducible factor-1 alpha; NEC, necrotizing enterocolitis.

To determine the role of HIF-1α in NEC-associated oxidative stress, we assessed the production of O_2_^•–^ and ROS, the protein carbonyl content, and the extent of lipid peroxidation. In the HIF-1α^loxP/loxP^ NEC mice, the levels of O_2_^•–^ and ROS, the protein carbonyl content, and MDA levels (a lipid peroxidation marker) increased significantly, and these effects were further augmented in the HIF-1α^ΔIEC^ mice (Figure 2A–D). Additionally, the HIF-1α^ΔIEC^ mice exhibited decreased levels of SOD, a major antioxidant enzyme (Figure 2E). The GSSG/GSH ratio, a representative indicator of the systemic redox status, increased significantly, resulting from a significant increase in GSSG and a decrease in GSH in the intestines of NEC mice, with a further elevation in the HIF-1α^ΔIEC^ mice (Figure 2F–H). These findings suggest that *Hif1a* knockout exacerbated NEC-induced oxidative stress.

### 3.3. IEC Proliferation Was Inhibited by HIF-1*α*^*Δ*IEC^

**Figure 3:**
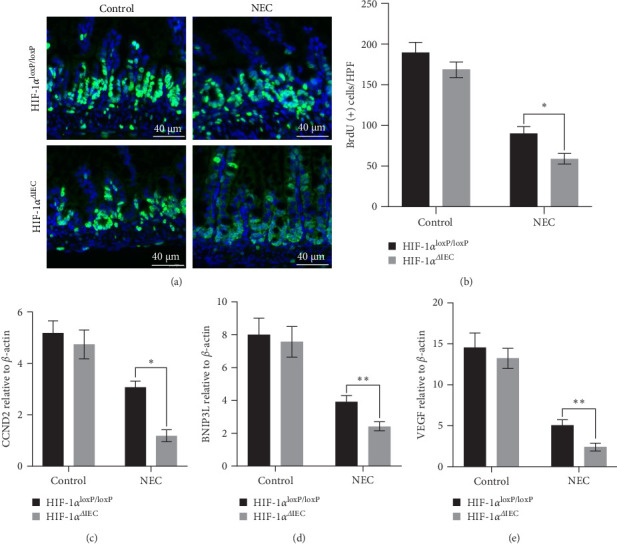
Evaluation of intestinal epithelial cell (IEC) proliferation: (A) BrdU immunostaining in the intestines. Scale bars: 40 µm. (B) Assessment of IEC multiplication (BrdU-positive cells per high-power field). (C–E) Detection of *Ccnd2*, *Vegf*, and *Bnip3l* mRNA levels using quantitative real-time reverse transcription polymerase chain reaction (RT-qPCR). Two-sided one-way one-way analysis of variance (ANOVA) was utilized for data comparison together with a post hoc Tukey test (*n* = 6–8, means ± the standard error of the mean [SEM]). *⁣*^*∗*^*p* < 0.05, *⁣*^*∗∗*^*p* < 0.01. HIF-1α, hypoxia-inducible factor-1 alpha; HPF, high power field; NEC, necrotizing enterocolitis.

To investigate the influence of HIF-1α^ΔIEC^ on IEC proliferation in the intestines, a crucial mechanism in NEC pathogenesis, we examined IECs using BrdU immunostaining. The proliferation of IECs was calculated based on the number of BrdU-positive cells. The proliferation of IECs was reduced significantly by NEC stress in the HIF-1α^loxP/loxP^ mice, and this effect was further aggravated in the HIF-1α^ΔIEC^ mice (Figure 3A,B). Additionally, the expression levels of HIF-1α target genes involved in cell survival, including *Ccnd2* (encoding cyclin D2), *Vegf* (encoding vascular endothelial growth factor), and *Bnip3l* (encoding BCL2 interacting protein 3 like), were further suppressed in HIF-1α^ΔIEC^ mice under NEC stress (Figure 3C–E). Collectively, these results indicated that HIF-1α^ΔIEC^ impaired cell proliferation in the context of NEC.

### 3.4. Increased IEC Apoptosis and Barrier Dysfunction in HIF-1α^ΔIEC^ Mice

**Figure 4:**
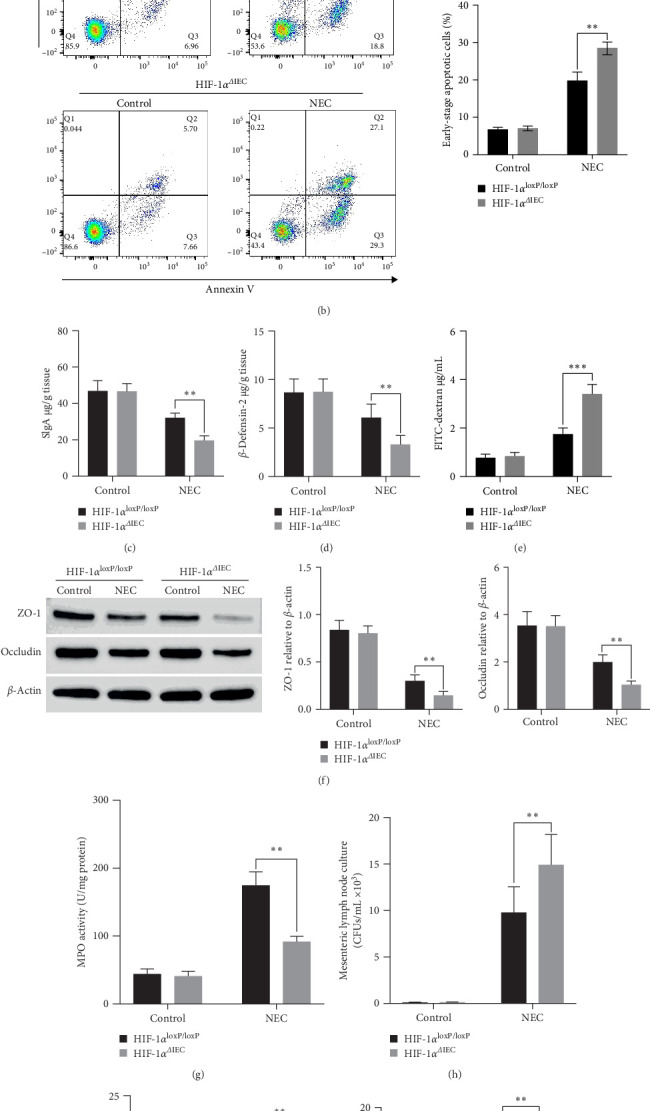
Evaluation of apoptosis and intestinal barrier injury: (A) western blotting analysis of caspase 3 and caspase 8 levels in mouse intestines. β-Actin comprised the loading control. Right panels: densitometry analysis of the immunoreactive protein bands. (B) Flow cytometry analysis of apoptosis in intestinal epithelial cells (IECs). The percentage of annexin-positive and propidium iodide (PI)-negative cells represents the cell apoptosis rate. Right panel: histogram analysis for multiple flow cytometry experiments. (C, D) Estimation of SIgA and β-defensin-2 levels in the terminal ileum. (E) Detection of serum fluorescein isothiocyanate (FITC)–dextran concentrations. (F) Western blotting analysis of ZO-1 and occludin levels in mouse intestines. Right panels: densitometry analysis of the immunoreactive protein bands. (G) Assessment of myeloperoxidase (MPO) activity in the ileum. (H–J) Quantification of bacterial growth in mesenteric lymph nodes, liver, and spleen. Data represent three independent experiments. Two-sided one-way analysis of variance (ANOVA) was utilized for data comparison together with a post hoc Tukey test (*n* = 6–8, means ± the standard error of the mean [SEM]). *⁣*^*∗∗*^*p* < 0.01, *⁣*^*∗∗∗*^*p* < 0.001. HIF-1α, hypoxia-inducible factor-1 alpha; NEC, necrotizing enterocolitis.

Apoptosis of IECs plays a crucial role in intestinal barrier dysfunction, contributing to the development of NEC. The HIF-1α^ΔIEC^ mice exhibited elevated levels of caspase 3 and caspase 8 compared with those in the HIF-1α^loxP/loxP^ mice under NEC conditions, indicating an increased level of apoptosis (Figure 4A). Additionally, the number of cells positive for annexin-V, a marker of apoptosis, was higher in the HIF1α^ΔIEC^ mice than in the HIF-1α^loxP/loxP^ mice under NEC stress (Figure 4B). We further evaluated the effects of HIF-1α^ΔIEC^ on intestinal barrier function by measuring SIgA levels, β-defensin-2 expression, FITC–dextran leakage, occludin, and tight junction proteins ZO-1. The HIF-1α^ΔIEC^ mice exhibited enhanced intestinal barrier injury induced by NEC stress compared with that in the HIF-1α^loxP/loxP^ mice (Figure 4C–F, Figure S1D). Moreover, under NEC stress, the HIF-1α^ΔIEC^ mice showed increased myeloperoxidase (MPO) activity, indicating heightened neutrophil infiltration (Figure 4G). Furthermore, we assessed the extent of bacterial translocation and found that the HIF-1α^ΔIEC^ mice experienced greater bacterial translocation compared with the HIF-1α^loxP/loxP^ mice, indicated by the bacteria culture of mesenteric lymph nodes, liver, and spleen (Figure 4H–J). These findings highlight the critical role of HIF-1α in the pathogenesis of NEC.

### 3.5. HIF-1*α*^ΔIEC^ Promotes Toll-Like Receptor 4 (TLR4)–Nuclear Factor Kappa B (NF-κB) Activation and the Inflammatory Response

**Figure 5:**
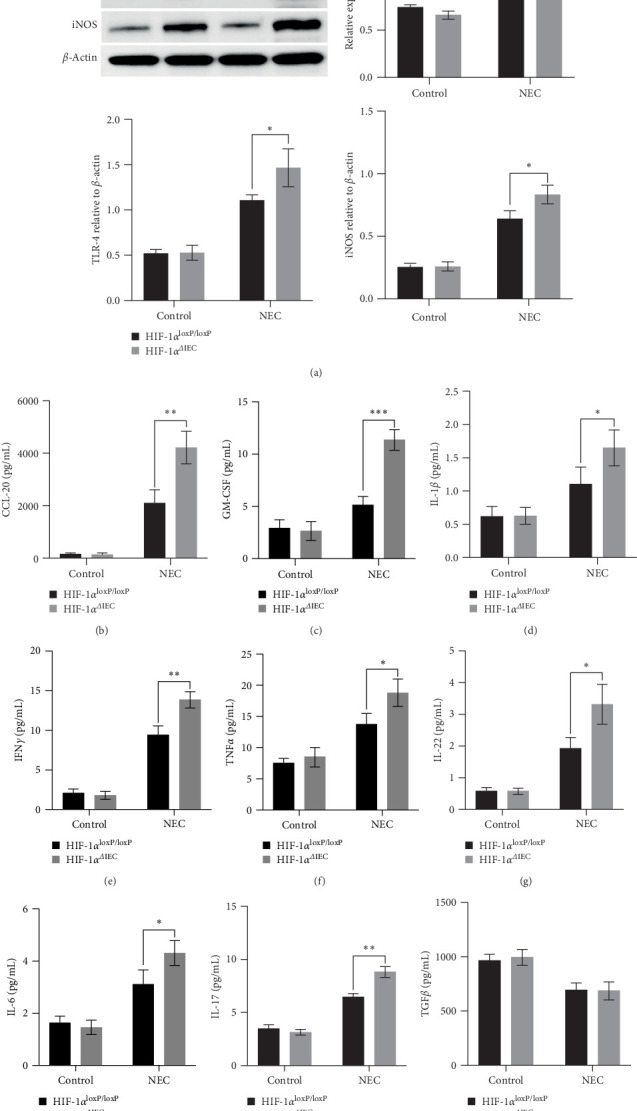
Evaluation of Toll-like receptor 4 (TLR4)–nuclear factor kappa B (NF-κB) activation and the inflammatory response: (A) western blotting analysis of p-p65, p65, TLR4, and inducible nitric oxide synthase (iNOS) levels in mouse intestines. Right panel: Densitometry analysis was performed. (B, C) Detection of CCL-20 and granulocyte macrophage-colony stimulating factor (GM-CSF) using enzyme-linked immunosorbent assay (ELISA) in the intestines. (D–I) Measurement of inflammatory cytokine concentrations in intestinal tissues using ELISA. Two-sided one-way analysis of variance (ANOVA) was utilized for data comparison together with a post hoc Tukey test (*n* = 6–8, means ± the standard error of the mean [SEM]). (J) The concentration of the anti-inflammatory cytokine transforming growth factor beta (TGF-β) in the intestines was detected using ELISA. *⁣*^*∗*^*p* < 0.05, *⁣*^*∗∗*^*p* < 0.01, *⁣*^*∗∗∗*^*p* < 0.001. HIF-1α, hypoxia-inducible factor-1 alpha; IFNγ, interferon gamma; IL, interleukin; NEC, necrotizing enterocolitis; TNFα, tumor necrosis factor alpha.

NEC stress led to an increase in TLR4–NF-κB pathway activation, as demonstrated by elevated levels of phosphorylated (p)-TLR4 and p-p65. Herein, the HIF-1α^ΔIEC^ mice exhibited further enhanced levels of p-TLR4 and p-p65 compared with those in the HIF-1α^loxP/loxP^ mice under NEC stress (Figure 5A). We also assessed the expression of inducible NO synthase (iNOS), a downstream target of NF-κB, and observed a robust induction of iNOS in the HIF-1α^ΔIEC^ mice under NEC stress (Figure 5A). In NEC mice, the levels of the cytokines CCL-20 and GM-CSF, both of which are regulated by NF-κB, were significantly higher in the HIF-1α^ΔIEC^ mice than in the HIF-1α^loxP/loxP^ mice (Figure 5B,C). Following NEC challenge, the levels of proinflammatory cytokines (interferon gamma [IFNγ], interleukin (IL)17, IL1β, IL22, IL6, and tumor necrosis factor alpha [TNFα]) increased, while the level of the anti-inflammatory cytokine transforming growth factor beta (TGFβ) decreased, in HIF-1α^loxP/loxP^ mice compared to controls (Figure 5D–J). Moreover, the HIF-1α^ΔIEC^ mice showed further increased levels of these proinflammatory cytokines, without any additional effect on the TGFβ level (Figure 5D–J). These findings suggested that HIF-1α^ΔIEC^ promotes TLR4–NF-κB activation and initiates an inflammatory response.

## 4. Discussion

Hypoxia is a crucial factor in the development of NEC, which is supported by various risk factors, such as birth asphyxia, congenital heart disease, and intrauterine growth restriction [20, 21]. The cellular response to hypoxia in the intestines is mainly modulated by HIFs [22], with HIF-1α being a conserved transcription factor that plays a role in the cellular adaptation to ischemia and hypoxia [23]. However, the specific role of HIF-1α in NEC development is unclear. Herein, we investigated the role and significance of HIF-1α in NEC pathogenesis. Through IEC-specific knockout of *Hif1a*, we observed increased intestinal damage in NEC mice, accompanied by oxidative stress, cell apoptosis, proliferation, and inflammation.

The gastrointestinal tract exhibits distinct variations in oxygen tension among mucosal tissues, which contribute to intestinal homeostasis and inflammation. HIFs have been implicated in regulating tissue metabolism, barrier function, inflammation, and immune responses in the intestines. Modulating hypoxia signaling has shown therapeutic potential in intestinal diseases [12]. For instance, the use of PHD inhibitors like DMOG can protect NEC mice by stabilizing HIF-1α [6]. These studies highlight the protective effects of HIF-1α in NEC.

The primary defense against pathogen invasion is IECs [24]. Impairment of the intestinal epithelium can lead to increased pathogen invasion, intestinal permeability, and intestinal damage, all of which are closely linked to the development of NEC [25, 26]. Given the role of HIF-1α in IEC physiology [9], we investigated the specific knockout of intestinal epithelial *Hif1a* in NEC mice. The results revealed exacerbated intestinal injury in HIF-1α^ΔIEC^ mice, which was not alleviated by DMOG treatment. These findings further emphasized the central role of HIF-1α in NEC pathology and support the results of a previous study that suggested its protective effect [6].

Oxidative stress has been implicated in NEC pathogenesis, with infants suffering from NEC exhibiting higher levels of oxidative stress compared with that of healthy controls [27]. Superoxide (O_2_^•–^) and ROS are commonly associated with oxidative stress. HIF-1α is closely linked to ROS, and its levels are increased in response to tissue hypoxia and increased ROS levels [28]. Previous studies have shown that HIF-1α can reduce ROS levels through various pathways during hypoxia [29, 30]. In line with these findings, the present study demonstrated that IEC-specific deletion of *Hif1a* increased oxidative stress in NEC mice, likely caused by impaired antioxidative capacity resulting from a lack of HIF-1α.

HIF-1α is vital for cell proliferation and viability because it regulates the expression of genes involved in metabolism and proliferation in response to low oxygen levels [31]. Our study revealed that HIF-1α^ΔIEC^ inhibited IEC proliferation in NEC mice, consistent with previous findings showing that stabilizing HIF-1α using DMOG enhanced IEC proliferation in NEC models [6].

The impact of HIF-1α on apoptosis can vary depending on the physiological conditions [32]. Herein, the specific knockout of *Hif1a* in IECs significantly increased apoptosis in NEC-stressed mice. The main barrier between the luminal and vascular compartments is the intestines, which is ensured by the presence of a mucus layer, adherent junctions, and tight junctions. Studies have demonstrated that HIFs provide protective effects to the intestinal barrier [10, 11, 33]. Indeed, mouse models with *Hif1a* knockout specifically in IECs or cell models with *Hif1a* knockdown exhibited mucosal barrier defects [12]. Similarly, our study showed more severe damage to the intestinal barrier in HIF-1α^ΔIEC^ mice. Collectively, these results highlight the crucial role of HIF-1α in protecting the intestinal barrier in NEC.

The TLR4–NF-κB signaling pathway is known to play a central role in NEC development [34], with increased TLR4 expression in the IECs of patients with NEC and NEC mice [35, 36]. TLR4 activation leads to NF-κB activation, resulting in excessive expression of proinflammatory cytokines, thus contributing to NEC pathology [34]. TLR4 can activate HIF-1α via NF-κB, and HIF-1α can also directly regulate TLR4–NF-κB signaling [37]. Building upon previous findings, our study showed that HIF-1α^ΔIEC^ further enhanced TLR4–NF-κB activation and exacerbated inflammation in NEC. Silencing HIF-1α has also been shown to dramatically enhance the NF-κB pathway signaling in the presence of LPS [38]. Moreover, HIF-1α activation has been demonstrated to reduce intestinal inflammation and protect against colitis in mouse models [39]. These findings suggest that HIF-1α-mediated inhibition of NF-κB and inflammation might serve as an important mechanism underlying the anti-inflammatory effects in NEC pathology.

There are some limitations to this study. First, HIF-1α is present in various cell types, such as endothelial cells and immunocytes; therefore, its significance in NEC protection across different target cell types remains unclear. Second, we only validated the role of TLR4–NF-κB signaling in HIF-1α^ΔIEC^ mice with NEC; thus, further research is needed to elucidate other signal pathways through which HIF-1α regulates intestinal injury and inflammation.

## 5. Conclusion

In conclusion, our study demonstrated the critical role of intestinal epithelial HIF1α in NEC pathology, particularly in relation to TLR4–NF-κB signaling. Therefore, regulation of HIF-1α might represent a potential therapeutic target to alleviate intestinal injury induced by NEC-related stress.

## Data Availability

The datasets generated during this study are available from the corresponding author on reasonable request.

## Supporting Information

Supporting Information Table S1 ELISA kits and primary antibodies used in this research. Table S2 Primer sequences used in the present study. Figure S1 (A, B) Western blotting and immunofluorescence analysis of intestinal HIF-1α expression in HIF-1α^loxP/loxP^ and HIF-1α^ΔIEC^ mice. (C) Gross morphology of the intestinal tissue samples. HIF-1α^ΔIEC^ mice exhibit more severe intestinal edema, inflammation, and erosion compared to HIF-1α^loxP/loxP^ mice under NEC stress. (D) Immunohistochemical staining of tight junction proteins ZO-1 in intestines. Student's *t*-test was used for the data comparison. Figure S2 DMOG alleviated NEC severity in HIF-1α^loxP/loxP^ mice but not in HIF-1α^ΔIEC^ mice.
