# Supplementary material for: Hypoxia-Inducible Factor-1α Modulates the Toll-Like Receptor 4/Nuclear Factor Kappa B Signaling Pathway in Experimental Necrotizing Enterocolitis
Source: Mediators Inflamm. 2024 Dec 17;2024:4811500. doi: 10.1155/mi/4811500 (PMC11668547; doi:10.1155/mi/4811500)
Supplement: Supporting Information — Table S1 ELISA kits and primary antibodies used in this research. Table S2 Primer sequences used in the present study. Figure S1 (A, B) Western blotting and immunofluorescence analysis of intestinal HIF-1α expression in HIF-1αloxP/loxP and HIF-1αΔIEC mice. (C) Gross morphology of the intestinal tissue samples. HIF-1αΔIEC mice exhibit more severe intestinal edema, inflammation, and erosion compared to HIF-1αloxP/loxP mice under NEC stress. (D) Immunohistochemical staining of tight junction proteins ZO-1 in intestines. Student's t-test was used for the data comparison. Figure S2 DMOG alleviated NEC severity in HIF-1αloxP/loxP mice but not in HIF-1αΔIEC mice. [file 4811500.f1.docx]

**Supplementary Materials and Methods**

**Methods**

**Assessment of reduced Glutathione (GSH) and Glutathione Disulfide (GSSG) levels**

Isolated intestinal tissues were homogenized and centrifuged at 10,000 × *g* for 10 min; 20 μL of the supernatant fraction was used for subsequent GSH and GSSG assessment. The samples were incubated with the respective assay buffer and probes from the GSH and GSSG Assay Kit (S0053, Beyotime, Shanghai, China) for 25 min at room temperature after thorough mixing. The fluorescence intensity (A_412_) was then measured. The total Glutathione and GSSG levels were calculated from their standard curve, respectively. GSH = Total Glutathione-GSSG × 2. The GSH and GSSG contents were expressed as nanomoles (nmol)/mg protein. The ratio of GSH/GSSG was regarded as an index for the oxidation level.

**Morphological assessment**

Intestinal tissue was fixed using 4% paraformaldehyde for 24 h, dehydrated, and embedded in paraffin. Then, the paraffin blocks with embedded tissues were cut into 4‑mm-thick sections using a rotary microtome. The sections were stained with hematoxylin and eosin (H&E) and then photographed under a microscope with a built‑in digital camera system. Two researchers, who were blinded to the treatment groups, used a standard histological scoring system to assess the intestinal injury, which was graded on a 5-point scale (grade 0 to grade 4), including: Grade 0: no injury; grade 1: injury to villus tips or colonic epithelium, or mild separation of the lamina propria; grade 2: mid‑villus disruption and/or moderate separation of the lamina propria; grade 3: complete villus disruption and/or severe separation and/or edema in the submucosa; and grade 4: transmural injury. Grade 2 or above was defined successful NEC.

**Annexin V/PI staining**

Fluorescein isothiocyanate (FITC)-annexin V and propidium iodide (PI) staining from a Cell Apoptosis Kit (Bestbio, Shanghai, China) was used to analyze apoptosis. Briefly, 1 × 10^6^ cells were collected at 1000g for 5 min at room temperature and washed twice using precooled phosphate-buffered saline (PBS). Cells were diluted with 500 μL of 1 × Binding Buffer and then stained with 20 μL of Annexin-V-FITC and 15 μL PI for 15 min protected from light at room temperature.

**Enzyme-linked immunosorbent assay (ELISA)**

Intestinal segments with equal weight (0.1 g) were collected, homogenized with PBS (1 mL), and centrifuged at 2500 rpm for 20 min at 4 ℃. Then, the supernatants were obtained and subjected to detection of granulocyte macrophage-colony stimulating factor (GM-CSF), C-C motif chemokine ligand 20 (CCL-20), and proinflammatory cytokines using ELISA kits. The concentrations of secretory immunoglobulin A (SIgA) and β-defensin-2 in the intestines were determined using ELISA kits based the corresponding protocols provided by manufacturers.

**Intestinal epithelial cell isolation and culture**

The resected small intestine was washed twice in sterile medium, cut into 1–2 mm pieces, digested in ethylenediaminetetraacetic acid solution (1.25% trypsin, 0.5 mmol/L) at normal temperature, and incubated with media that contained 200 U/mL collagenase type IV for 30 min at 37 °C. The digested tissue was centrifuged at 100 × *g* for 3 min and resuspended. Following filtration through a 100 μm cell-strainer, epithelial cells were washed twice and incubated at 37 °C and 5% CO_2_ in Dulbecco’s modified Eagle’s medium (DMEM) containing with 4.5 g/L glucose, 4 mmol/L L-glutamine, 1 mmol/L sodium pyruvate, 100 U/L bovine insulin, 1.5 g/L NaHCO_3_ and 10% fetal bovine serum.

**Western blotting analysis**

Snap-frozen terminal ileum tissues were homogenized, centrifuged, and the supernatant was collected. The protein content was calculated employing the bicinchoninic acid approach. Equal amounts of proteins (40 mg) were subjected to sodium dodecyl sulfate-polyacrylamide gel electrophoresis, transferred to polyvinylidene difluoride membranes (IPFL00010; Millipore, Burlington, MA, USA). The membranes were blocked using QuickBlock™ Western (Beyotime) and subsequently incubated overnight at 4 ℃ with primary antibodies. Following primary antibody incubation, membranes were treated with appropriate horseradish peroxidase-conjugated secondary antibodies for 1 hour at room temperature to visualize the immunoreactive protein bands. The optical densities of these bands were analyzed using ImageJ software (NIH, Bethesda, MD, USA).

**Reactive oxygen species (ROS) production assessment**

Intestinal sections were homogenized in sterile PBS (1 mL), and supernatants were collected post-centrifugation. The protein concentrations in the intestinal supernatants were measured using the bicinchoninic acid method. The levels of H_2_O_2_ in the intestinal supernatants were detected by adding respiratory substrates and Amplex Red, followed by incubation and measurement of the fluorescence intensity (A_570_).

**Detection of superoxide dismutase (SOD) and myeloperoxidase (MPO) activities, and malondialdehyde (MDA) levels**

Intestinal tissues were collected from mice, homogenized with sterile saline solution (0.1 g of each tissue with 0.9 mL of solution), and centrifuged at 14000 rpm for 10 min at 4 ℃. Then, the supernatants were collected for subsequent detection of SOD and MPO activities and MDA levels, according to the instructions of the following kits Malondialdehyde (MDA) assay kit (A003-1-2, Nanjing Jiancheng Bioengineering Institute, Nanjing, China); Superoxide Dismutase (SOD) assay kit (A001-3-2, Nanjing Jiancheng Bioengineering Institute); Myeloperoxidase assay kit (A044-1-1, Nanjing Jiancheng Bioengineering Institute)

**Bromodeoxyuridine (BrdU) immunostaining**

To measure intestinal epithelial cell proliferation, pups from different groups were intraperitoneally injected with 5-BrdU (50 mg/k; HY-15910, MedChemExpress, Monmouth Junction, NJ, USA) and sacrificed 18 h later. Samples of their terminal ileum were subjected to immunofluorescence staining using anti-BrdU antibodies.

| Product Name | Catalogue Number | manufacturer | Country |
| --- | --- | --- | --- |
| Mouse GM-CSF Quantikine ELISA Kit | MGM00 | R&D Systems | Minneapolis, MN, USA |
| Mouse CCL20/MIP-3 alpha Quantikine ELISA Kit | MCC200 | R&D Systems | Minneapolis, MN, USA |
| Mouse IL-1 beta/IL-1F2 Quantikine ELISA Kit | MLB00C | R&D Systems | Minneapolis, MN, USA |
| Mouse IFN-gamma Quantikine ELISA Kit | MIF00 | R&D Systems | Minneapolis, MN, USA |
| Mouse/Rat IL-22 Quantikine ELISA Kit | M2200 | R&D Systems | Minneapolis, MN, USA |
| Mouse TNF-alpha Quantikine ELISA Kit | MTA00B | R&D Systems | Minneapolis, MN, USA |
| Mouse IL-6 Quantikine ELISA Kit | M6000B | R&D Systems | Minneapolis, MN, USA |
| Mouse IL-17 Quantikine ELISA Kit | M1700 | R&D Systems | Minneapolis, MN, USA |
| Human/Mouse/Rat/Porcine/Canine TGF-beta 1 Quantikine ELISA | DB100C | R&D Systems | Minneapolis, MN, USA |
| β-Defensins ELISA Kit | hz-7481 | Shanghai Huzhen Biotechnology | Shanghai, China |
| SIgA ELISA Kit | HZA641Mu | Shanghai Huzhen Biotechnology | Shanghai, China |
| anti-β-actin antibody | 20536-1-AP | Proteintech | USA |
| anti-cleaved caspase 3 antibody | AF6311 | Affinity | USA |
| anti-cleaved caspase 8 antibody | AF6442 | Affinity | USA |
| anti-TLR4 antibody | AF7017 | Affinity | USA |
| anti-iNOS antibody | ab178945 | Abcam | Cambridge, UK |
| anti-p65 antibody | ab32536 | Abcam | Cambridge, UK |
| p-p65 | ab76302 | Abcam | Cambridge, UK |
| ZO-1 | AF5145 | Affinity | USA |
| Occludin | DF7504 | Affinity | USA |

**Table 1. ELISA kits and primary anti-bodies used in this research**

**Table 2.** Primer sequences used in the present study

| Gene | Forward sequence | Reverse sequence |
| --- | --- | --- |
| *Actb* | CATCCGTAAAGACCTCTATGCCAAC | ATGGAGCCACCGATCCACA |
| *Ccnd2* | GGGAACTGGTAGTGTTGGGTAAG | AATCATCGACGGCGGGTAC |
| *Vegf* | CAGATCATGCGGATCAAACCT | CTTTCTTTGGTCTGCATTCACATC |
| *Bnip3l* | TGAACAGCAGCAATGGCAATGAG | TTGTGGATGGAAGACGAGGAAGG |


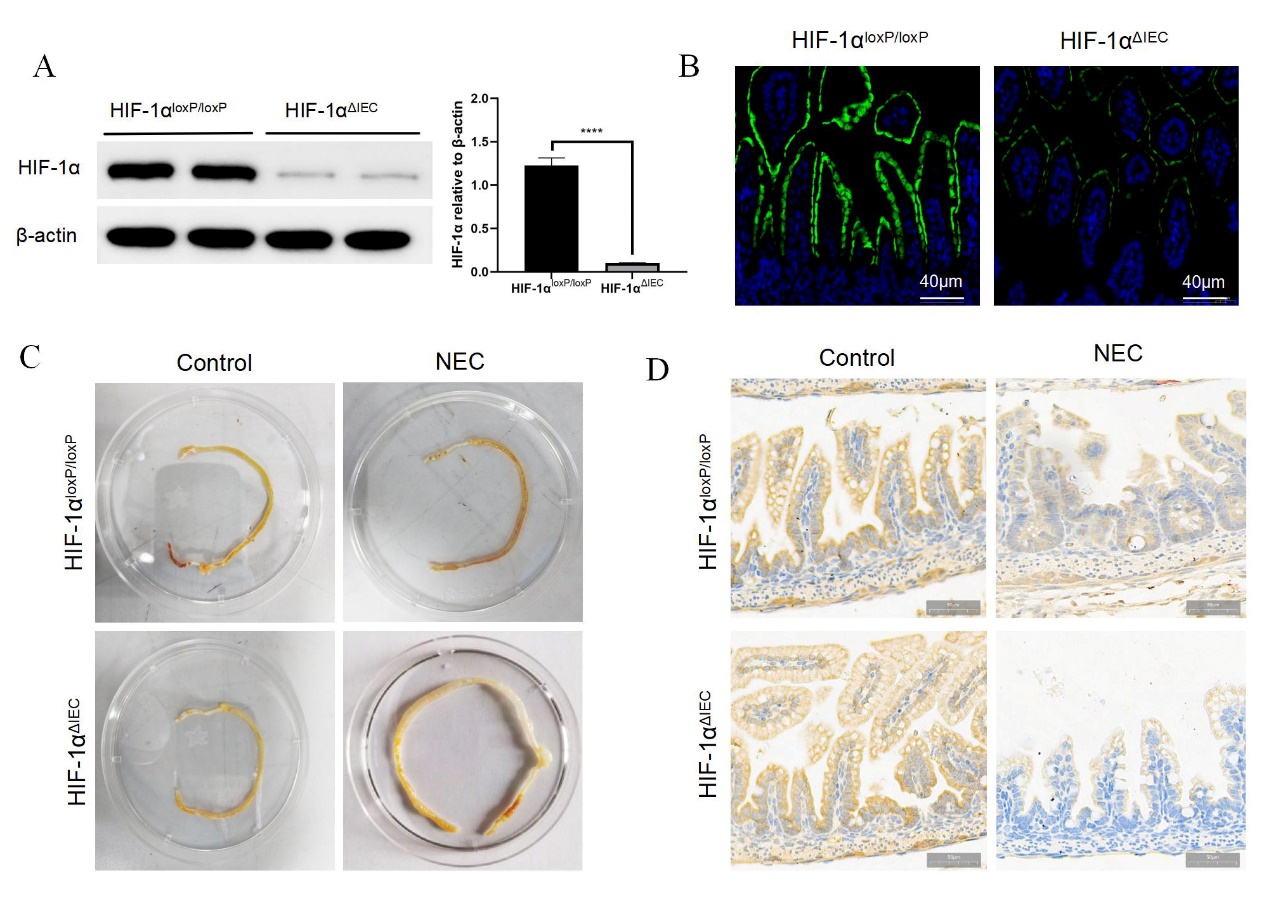


**Fig. S1.** (A, B) Western blotting and Immunofluorescence analysis of intestinal HIF-1a expression in HIF-1α^loxP/loxP^ and HIF-1α^ΔIEC^ mice. (C) Gross morphology of the intestinal tissue samples. HIF-1α^ΔIEC^ mice exhibit more severe intestinal edema, inflammation, and erosion compared to HIF-1α^loxP/loxP^ mice under NEC stress.  (D) Immunohistochemical staining of tight junction proteins ZO-1 in intestines. Student’s t-test was used for the data comparison.


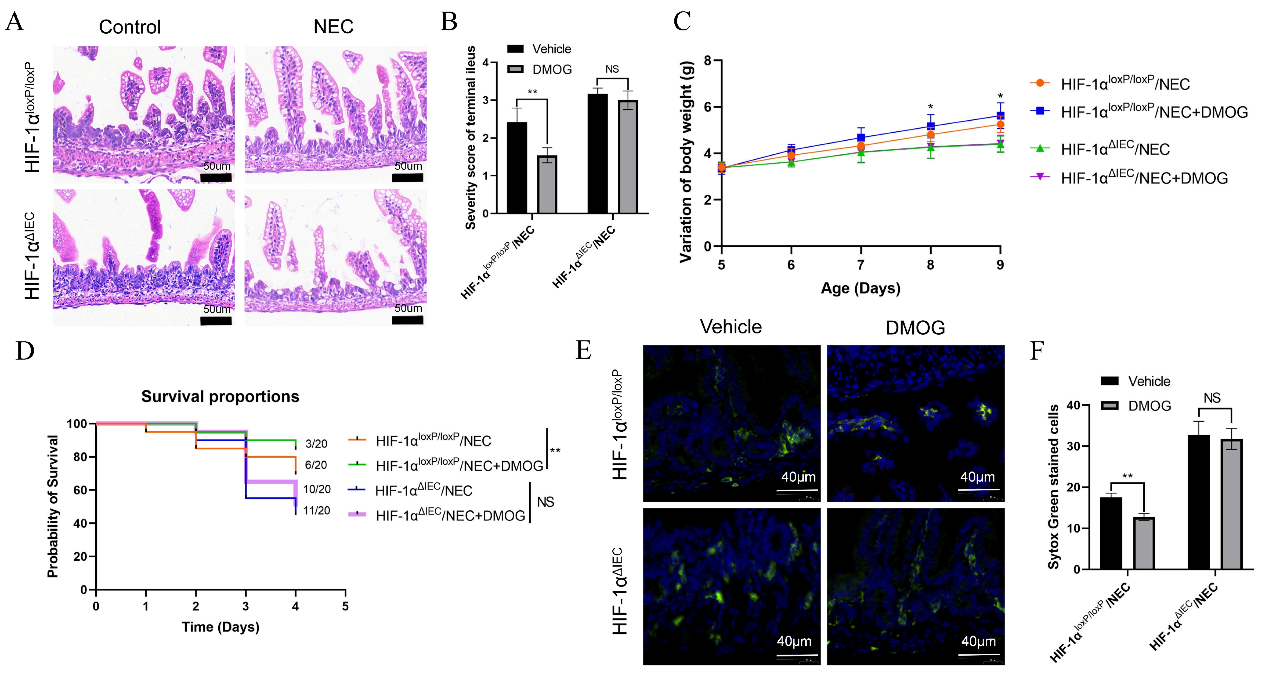


**Fig. S2. DMOG alleviated NEC severity in HIF-1α^loxP/loxP^ mice but not in HIF‑1α^ΔIEC^ mice.**

(A) Morphological detection. Scale bars: 50 μm. (B) Severity scores. (C) Weight changes. (D) Survival rates in the different groups. (E, F) Staining with the Sytox Green necrosis marker. Scale bars: 40 μm. Two-sided one-way ANOVA was utilized for data comparison together with a post-hoc Tukey test. (n = 8–20, means ± SEM). *P < 0.05, **P < 0.01.


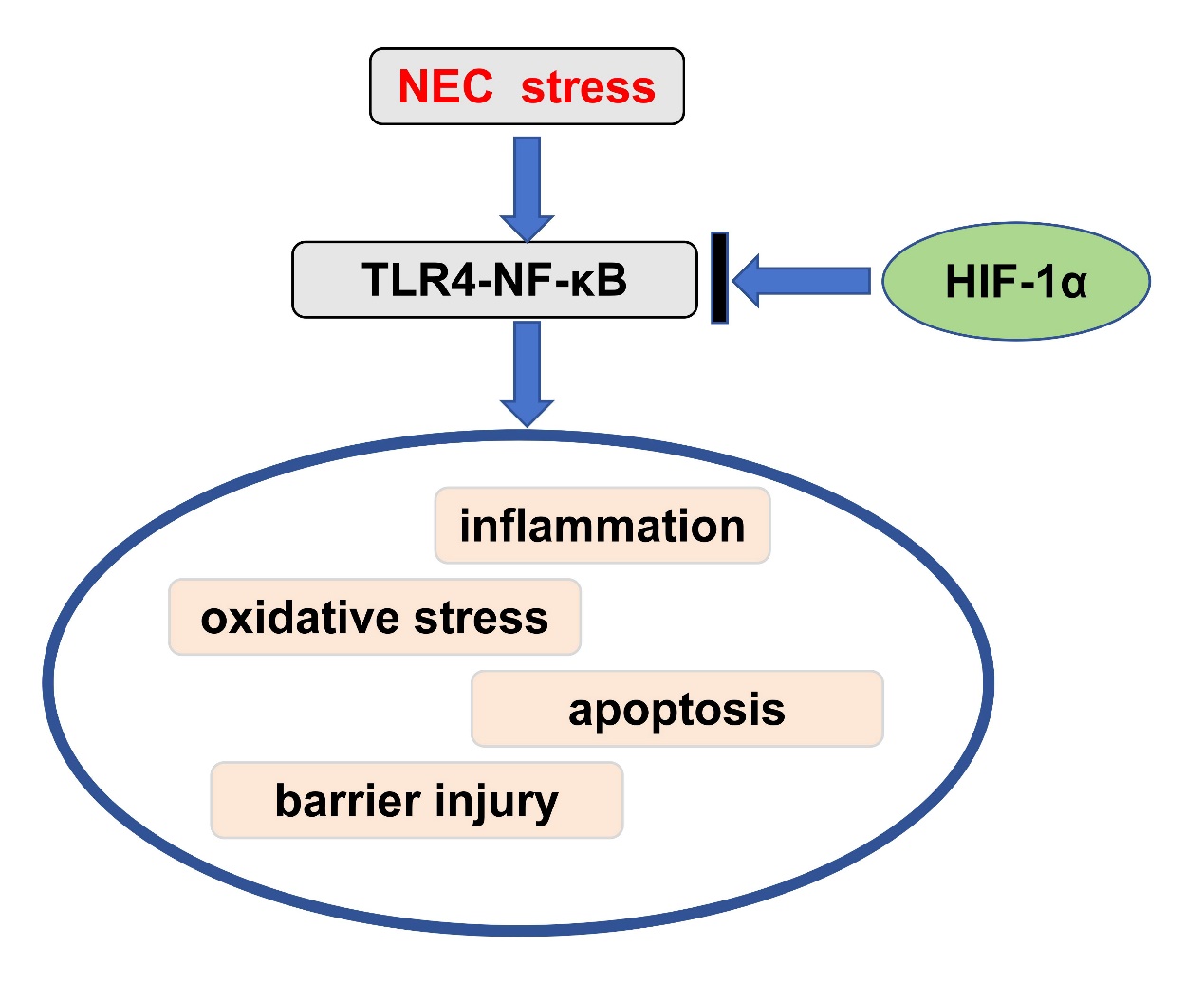


Graphical abstract
